# Dietary Supplementation of Attapulgite Improves Growth Performance in Pigs from Weaning to Slaughter

**DOI:** 10.3390/vetsci9100557

**Published:** 2022-10-11

**Authors:** Vasileios Kanoulas, Georgios A. Papadopoulos, Panagiotis Tassis, Georgia Koutouzidou, Georgios Arsenos, Paschalis Fortomaris

**Affiliations:** 1 Laboratory of Animal Husbandry, Faculty of Veterinary Medicine, Aristotle University of Thessaloniki, 54124 Thessaloniki, Greece; 2 Clinic of Farm Animals, Faculty of Veterinary Medicine, Aristotle University of Thessaloniki, 54124 Thessaloniki, Greece; 3 Institute of Plant Breeding and Genetic Resources, Hellenic Agricultural Organization-DIMITRA, 57001 Thessaloniki, Greece

**Keywords:** pigs, attapulgite, clays, growth, feed efficiency

## Abstract

**Simple Summary:**

Growth performance and feed efficiency of growing and fattening pigs is closely linked with gastrointestinal health. Τhe ban of using antibiotics as growth factors in the EU has directed research efforts towards alternative dietary and management tools to maintain pig performance. Clay minerals, and especially attapulgite, are among those materials with a variety of properties that can serve this purpose. The current study investigated the effects of the dietary supplementation of attapulgite on the performance of pigs from weaning to slaughter in three commercial pig farms in Greece. The results suggest that attapulgite dietary supplementation can improve pig growth and feed efficiency subject to continuous supplementation from the post-weaning period.

**Abstract:**

The objective of the present study was to investigate the effect of dietary supplementation of attapulgite on the performance of fattening pigs from weaning to slaughter under field conditions in three commercial farrow to finish herds. In total 1890 pigs were used for six months: 720 pigs in Farms A and B, respectively and 450 pigs in Farm C. The pigs were equally allocated in three dietary treatments: CON, standard diet in each growing phase; ATT, standard diet that was supplemented with attapulgite at 7 kg/tn of feed; and ATT+, standard diet that was supplemented with 8 kg/tn of feed with a compound product based on attapulgite. Pigs that were fed diets that were supplemented with attapulgite (ATT and ATT+) had significantly higher (*p* < 0.05) average daily feed intake (ADFI) and a better feed conversion ratio (FCR), compared to the control (CON). In conclusion, attapulgite supplementation in the diet of pigs from weaning to slaughter can improve their performance in commercial farms.

## 1. Introduction

The health and performance of growing pigs has been associated with gastrointestinal health and the composition of gut microbiota [1]. The latter is involved in nutritional, physiological, and immunological functions [1,2,3]. On the other hand, pathogens such as *Escherichia coli*, *Salmonella*, and *Clostridia* are also part of the intestinal microbial population in pigs [1]. These are opportunistic pathogens which can become pathogenic and cause diseases with substantial morbidity and mortality [1]. For this reason, antibiotics were frequently supplemented in pig diets to prevent these disturbances and hence reduce the risk of gastrointestinal disease [1]. The ban of antibiotic use as growth factors in the EU [4], led to intense research efforts for alternative dietary and management tools to maintain and improve pig growth performance. Various feed additives have been used as alternatives to antibiotics in pig production such as zinc oxide, copper sulphate, organic acids, enzymes, probiotics, prebiotics, essential oils, and clay minerals [5,6,7,8].

Attapulgite is a clay mineral that is used as a supplement in animal feed. Compared to other clays, attapulgite differs in structure because it lacks a continuous octahedral sheet structure [9]. Its unique structure is composed of ribbons of a 2:1 phyllosilicate, with each ribbon connected to the other one by the inversion of SiO4 tetrahedra along a set of Si-O-Si bonds [9,10]. This structure results in a fibrous morphology, with channels being parallel to fibers, this way resulting in a high specific surface area, high viscosity, and improved adsorption capacity [10,11]. Regarding its uses, previous studies have shown its ability to bind toxins in pigs and broilers [12] as well as its protective nature against diarrhoea in both animals and humans [13,14,15]. Chalvatzi et al. [16] reported improved laying hen performance after the inclusion of attapulgite in their diet. Recently, it was shown that attapulgite can be added to diets of LPS-challenged young broiler chickens to reduce inflammation and improve their antioxidant capacity [17]. Beneficial effects were also recorded for sow’s performance indexes following dietary supplementation of attapulgite in their diet [18,19].

Thus far, research concerning the effects of attapulgite supplementation in growing-fattening pigs has primarily focused on weaned piglets. Dietary supplementation of attapulgite improved growth performance and feed efficiency and reduced the incidence of diarrhoea in weaned piglets [20]. The supplementation of attapulgite in weaned piglet diets improved piglet growth performance and the apparent total tract digestibility of dry matter, gross energy, and crude protein [21,22]. The available literature regarding the use of attapulgite in pig diets during the overall fattening period is scarce. A recent study in a single pig herd investigated the effect of attapulgite supplementation together with benzoic acid on fattening pigs during a period of 2 months which included the fattening period from 50 kg to 100 kg [23]. It was shown that the growth performance and feed efficiency of pigs during the fattening stage were significantly improved by the co-supplementation of attapulgite with benzoic acid compared to those that were fed the unsupplemented diet [23]. To date, limited data are available on the effects of supplementation of attapulgite in pig diets on growth performance of pigs from weaning to slaughter. It is known that diseases of the gastrointestinal tract affect pigs from birth until they reach the market weight and hence impact the efficiency and profitability of pig herds [24]. Feed efficiency is a critical factor in pig production because feed production costs may reach up to 70% of the total production costs [25]. Although genetic selection has resulted in improved production levels, this evolution has also resulted in increased physiological demands, which may affect health and welfare at different growth stages [26]. Overall, based on its properties, it was hypothesized that attapulgite-enriched diets could lead to improved performance parameters at different growth stages from weaning to slaughter. The objective of the present study was to investigate the effect of attapulgite dietary supplementation on the performance of fattening pigs from weaning to slaughter under field conditions in three commercial farrow-to-finish pig herds.

## 2. Materials and Methods

### 2.1. Ethical Approval

The protocol was approved by the Research Committee of Aristotle University (Protocol No 67555/Code 85152) and followed the guidelines of Directive 2010/63/EU for animal experiments [27].

### 2.2. Study Material: Attapulgite

The attapulgite that was used in the present study was provided by Geohellas S.A., Athens, Greece. It is the same material that was used in our previously published research studies [18,19]. It was mined from deposits, located in Ventzia basin of Grevena, western Macedonia, Greece. Apart from the pure product that consisted of 75% attapulgite and 25% bentonite–saponite (Optify^®^, Geohellas S.A., Athens, Greece), the blend of attapulgite with other additives is further named as “attapulgite+” (ATT+). This product (Ultrafed^®^, Geohellas S.A., Athens, Greece) contained in wt.%: the pure attapulgite product (Optify^®^) 87.5%, *Saccharomyces cerevisiae* (Actisaf^®^ Sc47, strain CNCM I-4407, Lesaffre, Marcq-en-Baroeul Cedex, Lille, France) 1.56%, MgO 1.25%, lysine 1.25%, methionine 1.25%, palm oil 0.36%, NaCl 0.27%, vitamin and trace element supplements 1.00%, dextrose 3.60%, and enzymes (endo-1,4 β- glucanase & endo-1,4 β-xylanase) 1.66%.

### 2.3. Herds, Animals and Treatments

The study was performed in three farrow-to-finish farms (farms A, B, C) in central Greece with capacity of 820, 550, and 200 sows, respectively. In total 1890 pigs were used for a study period of six months. All animals derived from sows which were fed typical corn/barley/soy feeds (feed ingredient and nutrient analysis of the diets is available upon request from the corresponding author). The evaluation of 720 pigs from birth up to slaughter was performed in farms A and B. The animals were equally allocated in three groups (240 animals/group) namely group CON, ATT and ATT+. Each group consisted of 8 replicates/pens with 30 animals per replicate. In Farm B, each trial group consisted of 12 replicates/pens with 20 animals each. In Farm C, 450 pigs were included in the study. Grouping was the same as the other two farms, but in this case each group consisted of 150 animals with 15 pens/replicates of 10 animals/pen.

The main ingredient and nutrient composition of the diets during the nursery-weaning phase was: 58.5% corn, 12% soybean, 11% wheat bran, −18.8% crude protein (CP), 7.9% crude fat (CFat), 3.6% crude fiber (CF), 14.4 MJ/Kg metabolic energy (ME) (in Farm A); 34.8% corn, 15.0% wheat, 12.5% barley, 11.5% soybean meal, −18.9% CP, 4.1% CFat, 3.1% CF, 14.7 MJ/Kg ME (in Farm B); 58.5% corn, 14.0% soybean meal, 6.0% wheat bran, −17.7% CP, 4.2% CFat, 3.0% CF, 13.6 MJ/Kg ME (in Farm C). During the grower phase, the main ingredient and nutrient composition was: 55.4% corn, 25.5% soybean, 13.0% wheat bran, −17.8% CP, 5.8% CFat, 4.6% CF, 13.7 MJ/Kg ME (in Farm A); 31.0% corn, 20.0% barley, 15.0% wheat, 14.5% soybean meal, −19.2% CP, 4.0% CFat, 3.4% CF, 14.6 MJ/Kg ME (in Farm B); 27.0% wheat, 20.0% corn, 18.0% barley, 17.5% soybean meal,- 16.8% CP, 4.1% CFat, 4.0% CF, 13.5 MJ/Kg ME (in Farm C). During the finisher phase, the main ingredient and nutrient composition was: 56.0% corn, 23.5% soybean, 15.5% wheat bran, −17.2% CP, 5.2% CFat, 4.7% CF, 13.5 MJ/Kg ME (in Farm A); 9.9% corn, 25.0% barley, 35.0% wheat, 18.0% soybean meal, −17.4% CP, 2.7% CFat, 4.6% CF, 13.8 MJ/Kg ME (in Farm B); 27.0% wheat, 18.0% corn, 19.0% barley, 16.0% soybean meal, 15.0% wheat bran −15.3% CP, 3.3% CFat, 4.6% CF, 13.3 MJ/Kg ME (in Farm C). The soybean meal that was used in all diets contained 44.0% CP. The nutrient analysis of all diets was calculated with (calculated with EvaPig^®^ software; (version 1.4.0.0, Institut National de la Recherche Agronomique (INRA), Association Française de Zootechnie (AFZ) and Ajinomoto Animal Nutrition Europe, available from http://www.evapig.com/x-home-en; accessed on 30 June 2022).

### 2.4. Housing

Farm A consisted of five weaning units with a total capacity of 2035 weaners, four pre-fattening units with total capacity of 3250 animals, and three fattening units housing at maximum 3330 fatteners. A slatted floor was used in the weaning units and a semi-slatted floor was used in the pre- and fattening units. Farm B had two weaning units with a capacity of 12,900 animals, four pre-fattening units housing 1800 animals, and four fattening units with a capacity of 2000 animals. Farm C’s housing system included five weaning units with a maximum capacity of 540 weaners, one pre-fattening unit accommodating 750 animals and one fattening unit with maximum capacity of 750 animals. The temperature and ventilation were controlled automatically in all units and farms.

### 2.5. Parameters

The recorded parameters included body weight (BW) and average daily gain (ADG), feed consumption, feed conversion ratio (FCR), morbidity, and health parameters (scoring systems). BW measurements were performed on the day of weaning (25 ± 3 days of age), on the day of transfer to the pre-fattening unit (70 ± 5 days of age) and the fattening unit (112 ± 5 days of age), as well as at slaughter (170 ± 5 days of age). First, two weights were performed with an electronic scale with 50 g precision (Dini Argeo s.r.l., Via della Fisica n. 20, 41042 Spezzano di FioranoModena, Italia).

Feed consumption was measured on a weekly basis. The FCR was calculated as the ratio of feed (kg of feed consumed): growth (kg of body weight gain) in specific time periods and for the total study period. The ADG was calculated according to the following equation: ADG = Final weight–starting weight/days in the productive stage. Additionally, morbidity was recorded along with possible aetiology, whilst health parameters such as diarrhea score were also calculated upon examination of the pigs by the same veterinarian (V.K.). The veterinarian assessed diarrhea scoring based on a specific scoring scale at the completion of the first week post-weaning in each farm. The scoring scale is presented in Table 1.

### 2.6. Statistical Analysis

All statistical procedures were performed using SPSS (SPSS 25.0 Version, Chicago, IL, USA). As there were differences in the initial weight of the animals and in the duration of growth in each phase and in each farm, data regarding ADG, FCR, ADFI, and BW were analyzed using a single-factor between-subjects analysis of covariance (ANCOVA), with initial weight and duration of growth serving as covariates. In cases where the assumption of homogeneity of slopes was rejected (significant interaction between treatment and covariates was revealed) or the effect of both the covariates on the dependent variable was not significant; an analysis of variance (ANOVA) was applied. Differences between the mean values of specific treatment groups were evaluated using Bonferroni and Tukey test. Where assumptions about either the variability or the form of populations’ distribution were seriously violated, the Kruskal–Wallis nonparametric test was applied to evaluate treatment that was depended on differences, while differences between the mean values of specific treatments were evaluated using the nonparametric Wilcoxon rank sum test (Mann–Whitney U-test). It must be mentioned that the data for all the farms were analyzed using the mixed model, where the farm was included as a random component. Significance was declared at *p*-value ≤ 0.05, unless otherwise noted.

## 3. Results

The three pig herds participating in the study differed in size, housing conditions, diet ingredients, and plausibly on husbandry practices. Our intention was not to intervene with any aspect of their routine management and feeding practices. For this reason, attapulgite and its compound product were supplemented on top of the rations across growth phases without modifying any other ingredient. It can be argued that such an experimental approach may involve numerous factors differing between farms that may influence the outcome. To overcome this, we present the results for each pig herd separately, and when presented globally for all the farms the factor pig herd was included in the final model as a random factor.

The effect of treatments on growth performance parameters in Farm A are presented in Table 2. During the weaning-nursery phase, ADG and FCR were significantly higher for ATT+ compared to ATT (*p* = 0.001 and *p* = 0.026, respectively). The BW at the end of the weaning period was higher for ATT+ than ATT and CON, while it was also higher in CON than ATT (*p* < 0.001). During the grower phase, a significant improvement was noted for the FCR in both ATT and ATT+ compared to CON (*p* < 0.001), while the ending BW was higher in the CON than the other two groups (*p* = 0.026). At the finisher stage there was no difference between the treatments. The analysis of the performance for the overall period from weaning to slaughter revealed a lower ADFI in both the attapulgite-supplemented groups compared to CON (*p* < 0.001) which also corresponded to a favorable improvement of FCR in these groups than CON (*p* < 0.001).

The effect of the treatments on growth performance parameters in Farm B are presented in Table 3. During the nursery-weaning phase, the ADFI and FCR were lower in both ATT and ATT+ than CON (*p* < 0.001 and *p* = 0.001, respectively). At the grower phase ADG and ADFI were higher in both ATT and ATT+ than CON (*p* = 0.001 and *p* = 0.049 respectively). At the same period, the FCR was lower in ATT+ than CON, with FCR in ATT being intermediate (*p* = 0.013). At the finisher stage, the ADFI and FCR were higher in ATT+ compared to CON (*p* < 0.001 and *p* = 0.020 respectively). The final BW was also higher in ATT+ than CON and ATT, while it was higher in ATT than CON (*p* < 0.001). For the overall study period, the ADG was significantly higher in ATT+ than CON and ATT and in ATT than CON (*p* < 0.001), while ADFI was significantly higher in ATT+ than CON (*p* = 0.005).

The effect of treatments on the growth performance parameters in Farm C are presented in Table 4. No significant effect was noted during the weaning phase. At the grower phase, the FCR was lower in ATT+ than CON (*p* = 0.002), while the ending BW was higher in ATT+ than CON (*p* = 0.005). At the finisher phase, the ending BW was higher in ATT+ than CON (*p* = 0.001). When analyzing the results for the overall study period, the ADG was significantly higher in the ATT+ group compared to CON (*p* = 0.001).

The evaluation of the treatment effects on the combined results from all farms are presented in Table 5. No significant effect was noted during the weaning period. During the grower phase, the ADG was higher in ATT and ATT+ than CON, and in ATT+ than ATT (*p* < 0.001). The FCR at the same period was lower for ATT and ATT+ compared to CON (*p* < 0.001). The ending BW was higher in ATT+ than CON and ATT (*p* < 0.001). At the finisher phase, the ending BW was higher in ATT+ than CON (*p* = 0.001). For the overall period from weaning until the end of the fattening phase, the ADG was significantly higher in both ATT and ATT+ compared to CON, while it was higher in ATT+ than ATT (*p* < 0.001). The FCR for the overall period was lower in both ATT and ATT+ compared to CON (*p* = 0.042).

The results regarding the fecal consistency on a pen basis are presented for all farms in Table 6. According to the results, in Farm A the % of cases showing a normal fecal consistency score (score 1) was greater for the ATT and ATT+ groups compared to CON (*p* = 0.001). The % of cases showing score 2 was higher in CON than ATT and ATT+ (*p* = 0.001). In Farm B, no significant difference for fecal consistency was noted. In Farm C, the only difference between the treatments was noted for the % of cases showing score 4, being higher in CON than ATT and ATT+ (*p* = 0.007).

## 4. Discussion

The present study showed that dietary supplementation of attapulgite can affect pig growth performance from weaning to slaughter. In our view, the present study provides an adequate basis of data that can be used as a guideline for the future use of attapulgite in commercial pig diets. Thus far, attapulgite supplementation in pig diets was evaluated in controlled experiments that were mainly focused during the post-weaning period. To our knowledge, this is the first study that reports the application of attapulgite or any similar clay mineral on the feed of fattening pigs throughout their productive life.

The evidence in the available literature has shown that the dietary supplementation of attapulgite was mainly investigated in pigs during the post-weaning period. In the study of Lv et al. [22], the effects of dietary attapulgite supplementation on nutrient utilization was studied in weaned piglets aged 24 days for a 6-week experimental period. Similarly, Zhang et al. [20] have supplemented various levels of attapulgite in piglets aged 24 days for a 6-week period. Tang et al. [21] assessed the effects of attapulgite supplementation in weaned piglets aged 28 days for a 4-week period. Lv et al. [22] reported a growth-promoting effect of attapulgite since the average daily gain (ADG) of piglets was significantly improved. This increase in weight gain was accompanied by an increase in feed intake [22]. Likewise, attapulgite resulted in an improvement of ADG when it was supplemented at the lowest dosage that was tested [21]. In the study of Zhang et al. [20], apart from an improved feed conversion in favor of the attapulgite groups, no significant effect on ADG or feed intake was found. In our case, no consistent results on ADG were noted. Specifically, an improvement in ADG during the post-weaning period was observed only in Farm A and in the case of the attapulgite compound group. The absence of any significant effect on ADG in Farms B and C of any attapulgite group also influenced the absence of a significant effect when all the farms were evaluated together. This could be attributed to the fact that initial BW differences at weaning occurred between the groups. Indeed, piglets in the control group at the start of the experiment were numerically heavier than the attapulgite groups in Farm A, tended to be heavier in Farm B, and were significantly heavier in Farm C. These differences in initial weight appeared to be significant when the results from all the farms were evaluated. Even though during the statistical evaluation of the results the initial body weight was included as a covariate, it is plausible that these differences influenced attapulgite effects on ADG. It is known that weaning weight is highly correlated with postnatal growth potential [28]. Nevertheless, pigs can compensate earlier growth deficiencies at later stages [28].

The results of the fecal consistency scoring during the post-weaning period show that attapulgite supplementation could decrease the incidence of diarrhea. Specifically, in Farm A attapulgite supplementation increased normal fecal consistency (score 1), while it reduced nearly by half the cases with a score 2 compared to the control group. In addition, in Farm B the attapulgite supplementation led to no cases of severe fecal consistency (score 4) compared to the control group, while a tendency to improve normal fecal consistency (score 1) was also detected. However, in Farm B no significant effects of attapulgite on improving fecal consistency were observed. Possible differences between the farms regarding biosecurity measures and/or preventive antibiotic usage during the post-weaning period could account for the differences that were observed. In any case, the effects of attapulgite supplementation towards improving fecal consistency during the weaning period agree with previous findings. Zhang et al. [20] showed that the rate of diarrhea decreased with supplementation of 2000 mg/kg attapulgite in weaned piglets. Similarly, the diarrhea rate in pigs that were fed 1800 and 3000 mg/kg attapulgite was significantly reduced compared to the pigs that were consuming the control diet [21]. Another study also demonstrated that the diarrhea index decreased after supplementation of 2000 mg/kg attapulgite compared to the control-fed piglets [22]. Previous studies confirmed that feeding other type of clays to pigs such as smectite [29], montmorillonite [30], or clinoptilolite [31] reduced the incidence, severity, and duration of diarrhea [8]. Based on the review by Subramaniam and Kim [8], several mechanisms could be proposed by which clays may reduce the incidence of diarrhea in piglets. In brief, these may include water absorption in the gut thus resulting in firmer feces, an alteration of the microbial population in the gut to a more beneficial microflora, a possible physical effect on the cell membrane of the bacteria resulting in lysis of the membrane, absorption or detoxification of bacterial toxins, adherence to the gastrointestinal mucous membranes and reinforcement of the physical mucous barrier, and an increase in the number of Goblet cells in the small intestine. As in the present study, no analysis was performed regarding the effects on microbial gut flora and on gut histopathology; we can only speculate on potential mechanisms involved. Furthermore, our study was conducted under field conditions and possible supplementation of ZnO and/or antibiotics during the post-weaning period may occur. It is known that both ZnO and antibiotics still are supplemented to reduce the incidence of diarrhea. However, they may also influence other factors in the gut such as the microflora or gut histomorphology. In any case, the current results confirm the diarrhea ameliorating effects of attapulgite as described previously but need to be carefully extrapolated for future field applications.

The potential growth performance effects of attapulgite supplementation were noted during the growing (pre-fattening) phase. The most consistent effect was a significant improvement of the feed conversion ratio. This effect was more evident for the attapulgite compound product as it was detected across all the farms. Still, when examining the results from all the farms, the attapulgite “simple” product also proven to lower the FCR. Apart from FCR, attapulgite supplementation, when examined in overall results from all farms, led to a significant increase in the average daily gain during the growing period. As aforementioned, no published data existed thus far regarding the attapulgite effects on pigs’ performance during the growing or fattening period. Nevertheless, previous studies have shown that feeding other types of clay in growing pigs can improve performance [8]. Prvulovic et al. [32] showed an increase in weight gain and an improvement in FCR in growing pigs that were fed diets that were supplemented with clinoptilolite. Montmorillonite supplementation to growing pigs also improved weight gain, feed intake, and feed conversion ratio [33]. Elsewhere, growing pigs that were fed a diet that was supplemented with sericite had higher weight gain and better feed conversion ratio than those that were fed an unsupplemented diet [34]. On the other hand, other studies did not find any significant improvement of parameters during the growing phase following clay supplementation [31,35,36]. In particular, in Farm A, a significant improvement was noted in both attapulgite-supplemented groups which reached up to 17% compared to the control one. This magnitude in FCR reduction may be surprising, however, similar studies have reported such findings. For instance, in the study by Trckova et al. [37], a 45% increase in weight gain together with a 16.9% improvement in the feed conversion ratio in weaned pigs that were fed diets that were supplemented with 1% kaolin was found. Elsewhere, an almost 12% reduction of FCR after palygoskite supplementation has been previously observed in weaned pigs by Zhang et al. [20], whereas a 16.5% improvement of FCR has been previously attributed to xylo-oligosaccharide supplementation in nursery piglets [38], and an improvement exceeding 13% was previously described by Papaioannou et al. [31] after clinoptilolite addition in weaners feed. Similar to the above-mentioned large improvements, this has been repeatedly observed in several feed additives studies such as in fattening pigs by Anestis et al. [23] where an improvement of 15.5% was also observed after attapulgite and benzoic acid addition in feed. Several mechanisms could be proposed underlining this performance enhancement effect during the growing period. Firstly, attapulgite was shown to be beneficial to the intestinal integrity in weaned piglets, in terms of increasing villus diameters of the duodenum and ileum and villus height of the ileum [20]. These features of intestinal morphology are important indicators of health and reflect the digestive and absorptive capacity of the gut [20]. As attapulgite supplementation was supplied continuously in the present study, it can be hypothesized that there is a carry-over effect of the improved intestine function at the subsequent growing stage. Secondly, clays may reduce the speed of passage of feed along the digestive tract and prolong digestion [5,8,37]. Attapulgite was shown to improve nutrient digestibility, e.g., crude protein, energy [21], and digestibility of dry matter and gross energy in weaned piglets [22]. It is more likely that such digestive functions may be still improved during the growing phase based on the performance results of the present study. It is interesting to note that during the fattening period, attapulgite supplementation did not seem to exert the same beneficial effects as in the growing phase. Prvulovic et al. [32] also detected no improvement during the finishing period when pigs were fed clinoptilolite, although significant effects were evident during the pre-fattening period. In our case, the only significant effect was the increased slaughter weight that was observed in the compound attapulgite group. This could be a possible carry-over effect from the previous phase, since the ending BW at the growing phase was superior in the latter group when examining the results from all the farms.

Although the ATT and ATT+ groups contained the same quantity of attapulgite, the ATT+ treatment contained other supplements in lesser quantities such as enzymes and yeast. It is known that cell wall degrading enzymes such as glucanase and xylanase can reduce digesta viscosity [39], and hence influence the digestibility of nutrients. Such effects were evident in sow diets containing high viscosity cereals such as barley and wheat [40]. According to Broadway et al. [41], yeast-based products supplemented in pig diets may improve growth and performance by establishing and maintaining a healthy gastrointestinal tract, particularly in piglets. It is plausible that these additives could have had an additive effect to those that are exerted by the attapulgite alone and thus contributed to the improved performance of the ATT+ treatment. Yet, this field warrants further investigation.

## 5. Conclusions

In conclusion, the results of the present study indicate that when attapulgite was supplemented from weaning to slaughter it can improve performance parameters such as the average daily gain, feed conversion ratio, and slaughter weight. Due to the improvement of feces consistency, attapulgite could act preventive for diarrhea during the post-weaning period alongside with the use of other preventive measures, e.g., antibiotics or ZnO. As in the present study, attapulgite products were administered continuously from weaning to slaughter, and further research is needed to investigate whether intermittent supplementation could result in significant improvements as well.

## Figures and Tables

**Table 1:** The scoring scale that was followed for the scoring of fecal consistency during the first week of the post-weaning period on a pen basis.

Score	Fecal Consistency
1	Firm
2	Soft, spreads slightly
3	Very soft, spreads readily
4	Watery, liquid consistency

**Table 2:** The effect of the treatment on the growth performance characteristics of pigs in Farm A (*n* = 8/treatment).

	Treatment			SEM	*p*-Value
	CON	ATT	ATT+		
Phase I (nursery)					
Initial BW (kg)	8.08	7.97	7.51	0.28	0.290
ADG (g)	350.47 ^ab^	317.90 ^a^	379.93 ^b^	9.36	0.001
ADFI (kg)	0.661	0.643	0.681	0.01	1.000
FCR	1.89 ^ab^	2.04 ^a^	1.80 ^b^	0.05	0.026
Ending BW (kg)	21.79 ^a^	18.94 ^b^	23.56 ^c^	0.55	0.000
Phase II (grower)					
ADG (g)	594.16	598.95	626.49	18.92	0.451
ADFI (kg)	1.687	1.406	1.463	0.01	1.000
FCR	2.85 ^a^	2.36 ^b^	2.36 ^b^	0.11	0.000
Ending BW (kg)	51.21 ^a^	47.24 ^b^	47.64 ^b^	1.11	0.016
Phase III (finisher)					
ADG (g)	880.76	838.21	845.35	22.70	0.411
ADFI (kg)	2.351	2.475	2.441	0.050	0.456
FCR	3.00	2.79	2.73	0.09	0.123
Final BW (kg)	87.96	88.58	90.91	0.83	0.121
Overall					
ADG (g)	611.36	613.10	632.00	7.78	0.108
ADFI (kg)	1.666 ^a^	1.555 ^b^	1.537 ^b^	0.025	0.000
FCR	2.73 ^a^	2.53 ^b^	2.43 ^b^	0.04	0.000

SEM: Standard error of the mean. ^a,b,c^ Means with different superscripts differ significantly.

**Table 3:** The effect of the treatment on the growth performance characteristics of pigs in Farm B (*n* = 12/treatment).

	Treatment			SEM	*p*-Value
	CON	ATT	ATT+		
Phase I (nursery)					
Initial BW (kg)	6.87	6.74	6.26	0.19	0.060
ADG (g)	386.03	368.55	386.58	9.47	0.290
ADFI (kg)	0.684 ^a^	0.603 ^b^	0.593 ^b^	0.010	0.000
FCR	1.787 ^a^	1.654 ^b^	1.551 ^b^	0.038	0.001
Ending BW (kg)	22.06	31.26	22.09	0.38	0.290
Phase II (grower)					
ADG (g)	403.16 ^a^	515.82 ^b^	539.77 ^b^	18.69	0.001
ADFI (kg)	0.904 ^a^	1.035 ^b^	0.971 ^b^	0.039	0.049
FCR	2.249 ^a^	2.077 ^ab^	1.821 ^b^	0.066	0.013
Ending BW (kg)	36.52	38.01	39.39	1.60	0.472
Phase III (finisher)					
ADG (g)	932.58	915.74	926.90	17.65	0.761
ADFI (kg)	2.146 ^a^	2.250 ^a^	2.378 ^b^	0.030	0.000
FCR	2.351 ^a^	2.472 ^ab^	2.519 ^b^	0.029	0.020
Final BW (kg)	81.12 ^a^	84.11 ^b^	85.60 ^c^	0.30	0.000
Overall					
ADG (g)	608.43 ^a^	632.87 ^b^	644.99 ^c^	2.43	0.000
ADFI (kg)	1.346 ^a^	1.374 ^ab^	1.406 ^b^	0.013	0.005
FCR	2.223	2.174	2.168	0.019	0.118

SEM: Standard error of the mean. ^a,b,c^ Means with different superscripts differ significantly.

**Table 4:** The effect of the treatment on the growth performance characteristics of pigs in Farm C (*n* = 10/treatment).

	Treatment			SEM	*p*-Value
	CON	ATT	ATT+		
Phase I (nursery)					
Initial BW (kg)	9.01 ^a^	8.48 ^ab^	7.59 ^b^	0.41	0.031
ADG (g)	414.30	411.11	409.95	12.00	0.979
ADFI (kg)	0.807	0.865	0.811	0.016	0.146
FCR	1.97	2.14	1.99	0.06	0.302
Ending BW (kg)	25.42	25.05	24.52	0.70	0.690
Phase II (grower)					
ADG (g)	621.15	693.97	765.14	24.53	0.633
ADFI (kg)	1.519	1.657	1.668	0.063	0.059
FCR	2.601 ^a^	2.421 ^a^	2.122 ^b^	0.018	0.002
Ending BW (kg)	56.20 ^a^	59.78 ^ab^	62.23 ^b^	1.12	0.005
Phase III (finisher)					
ADG (g)	698.13	728.84	763.58	35.25	0.349
ADFI (kg)	2.099	2.024	2.160	0.079	0.244
FCR	3.061	2.798	2.872	0.151	0.342
Final BW (kg)	104.23 ^a^	109.74 ^ab^	115.70 ^b^	1.77	0.001
Overall					
ADG (g)	598.15 ^a^	633.51 ^ab^	670.31 ^b^	15.85	0.001
ADFI (kg)	1.590	1.608	1.669	0.049	0.707
FCR	2.664	2.545	2.502	0.079	0.253

SEM: Standard error of the mean. ^a,b^ Means with different superscripts differ significantly.

**Table 5:** The effect of the treatment on the growth performance of pigs in all farms (*n* = 30/treatment).

	Treatment			SEM	*p*-Value
	CON	ATT	ATT+		
Phase I (nursery)					
Initial BW (kg)	7.90 ^a^	7.65 ^ab^	7.03 ^b^	0.20	0.013
ADG (g)	387.73	378.11	381.96	9.77	0.771
ADFI (kg)	0.71	0.69	0.71	0.17	0.894
FCR	1.83	1.90	1.83	0.045	0.447
Ending BW (kg)	22.95	22.65	22.75	0.388	0.848
Phase II (grower)					
ADG (g)	507.98 ^a^	586.17 ^b^	663.23 ^c^	19.23	0.000
ADFI (kg)	1.274	1.305	1.414	0.042	0.061
FCR	2.493 ^a^	2.241 ^b^	2.126 ^b^	0.059	0.000
Ending BW (kg)	45.44 ^a^	47.17 ^a^	51.33 ^b^	0.87	0.000
Phase III (finisher)					
ADG (g)	834.13	835.93	854.03	15.15	0.606
ADFI (kg)	2.234	2.194	2.314	0.036	0.061
FCR	2.775	2.667	2.680	0.063	0.412
Final BW (kg)	88.53 ^a^	93.12 ^ab^	99.89 ^b^	2.02	0.001
Overall					
ADG (g)	605.29 ^a^	625.88 ^b^	652.40 ^c^	6.61	0.000
ADFI (kg)	1.504	1.494	1.543	0.020	0.214
FCR	2.486 ^a^	2.390 ^b^	2.373 ^b^	0.034	0.042

SEM: Standard error of the mean. ^a,b,c^ Means with different superscripts differ significantly.

**Table 6:** Treatment effects on the % of cases of fecal consistency score in the different scoring categories on the study of the farms as assessed at the first week post-weaning.

		Treatment			
	CON	ATT	ATT+	SEM	*p*-Value
Farm A					
Score 1	70.8 ^a^	83.6 ^b^	88.9 ^b^	2.13	0.001
Score 2	25.1 ^a^	13.6 ^b^	9.9 ^b^	1.81	0.001
Score 3	4.1	2.8	1.2	0.54	0.103
Score 4	0.0	0.0	0.0	0.0	1.000
Farm B					
Score 1	75.4	72.6	79.8	2.02	0.360
Score 2	14.6	19.9	15.6	1.33	0.205
Score 3	9.8	7.5	4.6	1.39	0.346
Score 4	0.0	0.0	0.0	0.0	1.000
Farm C					
Score 1	58.6	68.6	71.3	2.49	0.088
Score 2	24.6	22.0	20.6	1.64	0.616
Score 3	14.0	9.3	8.0	1.38	0.179
Score 4	2.67 ^a^	0.0 ^b^	0.0 ^b^	0.42	0.007

SEM: Standard error of the mean. ^a,b^ Means with different superscripts differ significantly.

## Data Availability

The data are available upon request.
